# Synthesis and Characterization of Hierarchical Zeolites Modified with Polysaccharides and Its Potential Role as a Platform for Drug Delivery

**DOI:** 10.3390/pharmaceutics15020535

**Published:** 2023-02-05

**Authors:** Agata Wawrzyńczak, Izabela Nowak, Natalia Woźniak, Jagoda Chudzińska, Agnieszka Feliczak-Guzik

**Affiliations:** Faculty of Chemistry, Adam Mickiewicz University in Poznań, 61-614 Poznań, Poland

**Keywords:** hierarchical zeolites, mesoporosity, modification, polysaccharides, ibuprofen, curcumin, ferulic acid

## Abstract

Hierarchical zeolites are aluminosilicates with a crystal structure, which next to the micropores possess secondary porosity in the range of mesopores and/or small macropores. Due to their ordered structure and additional secondary porosity, they have aroused great interest among scientists in recent years. Therefore, the present work concerns the synthesis and characterization of hierarchical zeolites with secondary mesoporosity, based on commercial zeolites such as MFI (ZSM-5), BEA (β) and FAU (Y), and modified with polysaccharides such as inulin, hyaluronic acid, and heparin. All materials were characterized by various analytical techniques and applied as a platform for delivery of selected drug molecules. On the basis of X-ray diffraction (presence of reflections in the 2θ angle range of 1.5–2.5°) and low-temperature nitrogen sorption isotherms (mixture of isotherms of I and IV type) additional secondary porosity was found in the mesopore range. Additional tests were also conducted to determine the possibility of loading selected molecules with biological activity into the aforementioned materials and then releasing them in the therapeutic process. Molecules with different therapeutic options were selected for testing, namely ibuprofen, curcumin, and ferulic acid with anti-inflammatory, potentially anticancer, antioxidant, and skin discoloration activities, respectively. Preliminary studies have confirmed the possibility of using hierarchical zeolites as potential carriers for bioactive molecules, as the loading percentage of active substances ranged from 39–79% and cumulative release for ibuprofen reached almost 100% after 8 h of testing.

## 1. Introduction

In the last few years synthesis, characterization and application of hierarchical zeolites have become an object of interest for a growing number of scientists. In addition to micropores, hierarchical zeolites are characterized by having a secondary porosity in which there are pores of diverse sizes, i.e., supermicropores, mesopores or macropores [1].

Hierarchical modification of zeolite is assumed to reduce steric hindrance by introducing secondary porosity (mesopores and macropores). This allows the particle sizes of the reactants to be increased to a range that is needed for a given reaction. There are two types of active sites in materials with secondary porosity: active sites accessible from the external surface through secondary channels to the entrance of the microporous network (they have almost no steric hindrance), and active sites located at the entrance to the microporous network (they potentially have several steric limitations and limit accessibility for large molecules) [2].

The contribution and properties of secondary porosity, i.e., specific surface area, pore size and distribution, as well as pore volume, depend on the method of synthesis of hierarchical zeolites. So far, many diverse methods of obtaining hierarchical zeolites have been described, therefore, their classification may be a challenging task. Generally, the synthesis procedures for obtaining hierarchical zeolites can be divided into two main groups, namely bottom-up and top-down [2]. Bottom-up includes the following methods: hard templating, soft templating, non-templating, zeolization of materials or assembly of nanometer-sized zeolites [3]. On the contrary, top-down methods consist of dealumination, desilication, or recrystallization [2,4,5]. In general, the variety of methods developed to date for synthesis of hierarchical zeolites relies on aggregation, extraction, and crystallization processes [6].

Despite the large number of hierarchical zeolites available today, they can be divided, according to their origin and secondary porosity, into pure zeolitic phases and composites. Pure zeolitic phases or so called true hierarchical zeolites are obtained when the secondary porosity is localized within the zeolitic phase. In this case, the secondary porosity is present either within the zeolite crystals or in the intercrystalline spaces. In the case of composites comprising a zeolitic phase and a nonzeolitic phase the hierarchy in zeolites is due to additional phases. The nonzeolitic phase typically serves as a carrier or binder that holds the zeolite crystals together. When this phase is the carrier, secondary porosity is derived from the carriers [3]. 

In this study selected polysaccharides were used to modify hierarchical zeolites based on commercial materials such as MFI (ZSM-5), BEA (β), and FAU (Y). Polysaccharides, also known as complex sugars, are polymeric compounds with the following general formula (C_6_H_10_O_5_)_n_, placing them in the group of macromolecular compounds. The polysaccharide chain can take a simple (linear) or branched form. Complex sugars formed on the basis of one type of monosaccharides are called homopolysaccharides (homoglycans). These are, for example, starch, cellulose, and glycogen. On the other hand, polysaccharides composed of various types of monosaccharides can be found under the name of heteropolysaccharides (heteroglycans). They include, among others, hyaluronic acid, heparin, and inulin, namely polysaccharides used in the course of this work [7].

**Figure 1:**
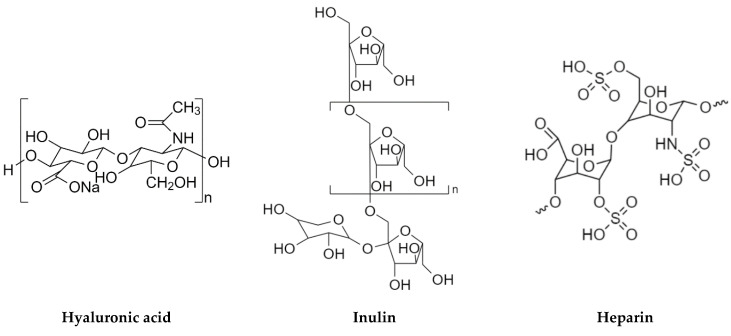
Structural formula of hyaluronic acid, inulin, and heparin.

Hyaluronic acid (HA), classified as a glycosaminoglycan (GAG), was first isolated from the vitreous body of the cow’s eye by Karl Meyer and John Palmer in 1934 [8,9]. Despite the presence of the word “acid” in the name it is not an acid but an unbranched biopolymer composed of repeating disaccharides that include D-glucuronic acid and N-acetyl-D-glucosamine (Figure 1) [10,11]. The bonds in the HA molecule are alternating β-1,3 and β-1,4-glycosides [10,11]. Both compounds are spatially related to the glucose molecule, which in the beta configuration allows all of its bulky groups to be in sterically favorable equatorial positions, while all small hydrogen atoms occupy sterically less favorable axial positions. Therefore, the disaccharide structure is energetically very stable [12].

HA is one of the most hydrophilic (water-loving) molecules in nature and can be referred to as a natural moisturizing agent [12]. Thanks to its ability to bind water, hyaluronic acid swells so that the tension state of the extracellular matrix increases in direct proportion to the volume of water taken up. Due to this property, it is very often used in the cosmetic industry [8,13,14]. Moreover, in physiological solution the HA backbone stiffens due to a combination of the chemical structure of the disaccharide, internal hydrogen bonds, and interaction with the solvent. Therefore, hyaluronate solutions exhibit very unusual rheological properties. [8,12,13,14].

Inulin is a polysaccharide composed of glucose and fructose molecules [15]. In 1804, it was first isolated from the perennial plant *Inula helenum* (i.e., Oman the Great of the Asteraceae family), by Valentin Rose, a prominent German researcher [16]. The inulin chain is linear (Figure 1), with fructose molecules joined by β-1,2-glycosidic bonds. At the end of the chain there is a glucose molecule. The number of fructose units is determined by the origin and the harvest time of the plants in which inulin is present, and varies from 10 to 70 [17]. This polysaccharide occurs in the form of a white powder that dissolves well in warm water, while it precipitates from a solution at 0 °C [18].

Inulin’s unique and flexible structure and its stabilizing and protective effects make it an excellent polysaccharide with a wide range of applications. Three hydroxyl groups attached to each fructose unit serve as an anchor for chemical modification. This in turn helps to increase its bioavailability, improve cellular absorption, and achieve long-lasting, targeted effects [19,20]. 

Heparin (Figure 1) was isolated from canine liver cells by Jay McLean. It is an organic compound of the polysaccharide group composed of mucopolysaccharides and sulfonated glycosaminoglycans. The structure of heparin is linear and is composed of polymers in the range of 3000 to 30,000 Daltons including monosaccharides. Heparin molecules contain outward-facing anionic groups with a negative charge [21,22].

Heparin is a natural anticoagulant that counteracts intravascular blood clotting. In this case mechanism of action includes stimulation of antithrombin, clotting factor IXa, and Xa, and reduction of aggregation and adhesion of thrombocytes [23]. 

To the best of our knowledge, hierarchical zeolites modified with heparin, inulin, or hyaluronic acid have not been obtained up to date. Generally, there is little work involving the synthesis of porous materials modified with the aforementioned compounds. Examples of the synthesis of such materials were published by Ojeda et al. [24] and Ari and Sahiner [25]. The first group proposed the synthesis of metal-modified mesoporous starches using a simple microwave-assisted method involving gelation of the parent polysaccharide and addition of the metal precursor, followed by solvent exchange and drying [24]. Paper by Ari and Sahiner reported chemically crosslinked superporous inulin cryogels made by cryogelation using divinyl sulfone, and ranging from 75% to 150% of inulin repeating units [25].

The use of polysaccharide-based systems as drug carriers could be profitable due to the high diversity of polysaccharides and their natural origin. They can create biocompatible and biodegradable systems with a broad range of both biological and chemical function-alternatives. These primarily include protection of therapeutic agents by by-passing the reticuloendothelial system, stabilizing biomacromolecules, and enhancing the bioavailability of incorporated small-molecule active ingredients. Therapeutic transport is a key to the utility of polysaccharide molecules: they move drugs from the site of administration to specific tissues through binding and mucosal transport and by using chemical, size and receptor targeting [26,27]. So far, polysaccharide-based materials were used as carriers in the combination with: hydrogels [28,29], zeolites [30] or lipid nanoparticles [31].

The interest in bioactive compounds is related to their potential use as food, chemical, cosmetic, or pharmaceutical additives [32]. In the present study, ibuprofen, curcumin and ferulic acid were used as bioactive compounds. Ibuprofen, which belongs to the group of non-steroidal anti-inflammatory drugs (NSAIDs) exhibits analgesic, antipyretic and anti-inflammatory effects. Curcumin is a natural medicinal substance belonging to the group of polyphenols, showing anti-inflammatory effects and potentially anticancer properties [33,34]. In turn, ferulic acid, which belongs to the group of phenolic acids, shows antioxidant and anti-inflammatory activity [35,36].

A prerequisite for effective and beneficial therapy is the delivery of a biologically active agent to the appropriate site in the body and its release, preferably in a controlled manner [37]. One way to release the active ingredient in a controlled manner is by using carriers of active ingredients [38,39]. To date, the following have been used as carriers of the active substance: carbon materials [40], polymeric materials [41] and, increasingly, porous materials such as zeolites [42,43].

The purpose of the presented work was to assess the suitability and possibility of using hierarchical zeolites based on commercial zeolites such as MFI, FAU and BEA modified with polysaccharides as carriers of active substances: ibuprofen, curcumin, and ferulic acid.

## 2. Materials and Methods

### 2.1. Synthesis of Hierarchical Zeolites

#### 2.1.1. Synthesis of Unmodified Hierarchical Zeolites Based on Zeolite MFI (ZSM-5), BEA (β) or FAU (Y)

The preparation of unmodified hierarchical zeolite MFI (ZSM-5), BEA (β) or FAU (Y) was based on dispersing weighed amount (0.75 g) of commercial zeolite of ZSM-5 (Acros Organics B.V.B.A., Geel, Belgium), β (Alfa Aesar, Ward Hill, MA, USA) or Y (Alfa Aesar, Ward Hill, MA, USA) type in a mixture containing 150.00 g of distilled water, 1.875 g of ammonia (StanLab, Lublin, Poland), 90.00 g of ethanol (StanLab, Lublin, Poland), and 0.525 g of cetyltrimethylammonium bromide (CTABr) (Fluka Analytical, Buchs, Switzerland). This process was carried out in a polyethylene bottle, for a period of 30 min, at 65 °C, using an ultrasonic bath. After 30 min, 0.84 g of tetraethyl orthosilicate (TEOS) (Aldrich Chemistry, Saint Louis, MO, USA) was added to the solution as a source of silicon. The entire solution was then stirred on a magnetic stirrer for 4 h at 65 °C.

After this time, the precipitate obtained was filtered on a glass funnel using a filter paper, washed with a mixture of distilled water and ethyl alcohol in a volumetric ratio of 1:1, and left to dry in air at room temperature. After drying, the precipitate was calcined in order to remove the templating agent (CTABr). The calcination was carried out for 5 h at 550 °C.

#### 2.1.2. Synthesis of Hierarchical Zeolites Modified with Inulin

The synthesis of hierarchical inulin-modified zeolites based on commercial zeolites such as MFI, BEA, or FAU initially proceeded in a manner similar to that of the synthesis of pure hierarchical zeolites. However, inulin (Chemat, Gdańsk, Poland) was added as a modifying compound in the amount of 0.05 g together with 0.84 g of the silicon source (TEOS). Thereafter, the course of the reaction was similar. An extraction process was used to remove template (CTABr) from inulin-modified materials. For the extraction process 0.50 g of the obtained material was mixed with 50.00 mL of ethanol and 0.50 mL of HCl solution. The whole mixture was stirred on a magnetic stirrer for 24 h at 65 °C using a reflux condenser. The precipitate was filtered on a glass funnel equipped with filter paper, washed with ethanol, and dried in air at room temperature.

#### 2.1.3. Synthesis of Hierarchical Zeolites Modified with Hyaluronic Acid

The process of synthesis of hierarchical zeolites based on commercial zeolites type ZSM-5, Beta, or Y, modified with hyaluronic acid, was similar to that of hierarchical materials modified with inulin (Section 2.1.2), only when adding 0.84 g TEOS, 0.05 g of hyaluronic acid (Chemat, Gdańsk, Poland) was added instead of inulin. The remaining course of the synthesis of hyaluronic acid-modified materials was analogous to that described above.

#### 2.1.4. Synthesis of Hierarchical Zeolites Modified with Heparin

The process of synthesis of hierarchical zeolites based on commercial types of zeolites such as ZSM-5, β, or Y, modified with heparin, was analogous to the synthesis of materials described in Section 2.1.2 and Section 2.1.3, only during the addition of 0.84 g of TEOS, 0.05 g of heparin (Biosynth Carbosynth, Staad, Switzerland) was added instead of inulin or hyaluronic acid. The remaining course of synthesis of heparin-modified materials, was analogous to those described above.

### 2.2. Designation of Materials Used in the Work

Table 1 shows the designations of the materials synthesized and characterized in this paper.

### 2.3. Characterization of the Obtained Hierarchical Zeolites

The hierarchical zeolites synthesized were subjected to physicochemical characterization by the following techniques:

- Elemental analysis;
- X-ray diffraction (XRD);
- Low-temperature nitrogen adsorption/desorption measurements;
- Transmission electron microscopy (TEM)

#### 2.3.1. Elemental Analysis

The determination of the elemental composition of the synthesized catalysts was performed in the Department of Chemistry, Adam Mickiewicz University in Poznań, with the use of the Vario EL III (Elementar Analysensysteme GmbH, Langenselbold, Germany) elemental analyzer apparatus. The measurement method involves the catalytic combustion of the sample (10–20 mg) at 1200 °C and analysis of the composition of combustion gaseous products, which is based on differences in their thermal conductivity.

#### 2.3.2. XRD—X-ray Diffraction

X-ray diffraction studies were performed using a Bruker AXS D8 Advance (Bruker, Billerica, MA, USA) diffractometer with a Johannson monochromator and a LynxEye stripline detector. The CuKα radiation source generated a wavelength of λ = 0.154 nm. Measurements were made in the low angle range of 2ϴ = 0.6–8.0° (with 0.02° accuracy) and in the high angle range of 2ϴ = 6.0–60.0° (with 0.05° accuracy).

#### 2.3.3. Low-Temperature Nitrogen Adsorption/Desorption Measurements

The measurements were performed using a Quantachrome Autosorb iQ apparatus (Quantachrome Instruments, Boynton Beach, FL, USA). Prior to the actual measurement, the samples were degassed under vacuum at 110 °C for 24 h. The sorption isotherms were obtained at −196 °C, in the relative pressure range p/p_0_ from 0.02 to 1.00.

#### 2.3.4. Transmission Electron Microscopy (TEM)

Microscopic images of the materials were taken using a transmission electron microscope JEOL JEM-1200 EX II (JEOL, Akishima, Tokyo, Japan) operating at 80 kV.

### 2.4. Active Substances Loading

250 mg of the carrier (functionalized hierarchical material) was poured with 5.00 mL of ethanol (99.8%, POCh) and then 150 mg of the active substance (ibuprofen, curcumin or ferulic acid) were added. The suspension was stirred on a magnetic stirrer for 24 h. After this time, the whole was filtered and the product obtained was air-dried. Once the loading of the active substance to the hierarchical materials was completed, the loading percentage of the active substance on the carrier used was calculated (% LOAD). The efficiency of the loading of the active substance in the hierarchical zeolite structure was calculated on the basis of Equation (1).

(1) $$\%\;\mathrm{LOAD}=\frac{\mathrm{initial}\;\mathrm{amount}\;\mathrm{of}\;\mathrm{active}\;\mathrm{substance}\;\left[g\right]}{\mathrm{mass}\;\mathrm{of}\;\mathrm{the}\;\mathrm{complex}\;\left(\mathrm{active}\;\mathrm{substance}+\mathrm{carrier}\right)\left[g\right]}\times 100\%.\;$$

Equation (1) is a formula for the calculations of the percentage of active substance loading on the carrier [44].

### 2.5. Release Profiles of Active Substances

20 mg of the carrier loaded with an active substance was weighed into the vial and the mixture of 20.00 mL of phosphate buffer (pH 5.8) and 5.00 mL of permeation promoter (glycerin or ethanol) was added. The process was carried out at room temperature for 24 h with absorbance measurements of the released active substance performed every 30 min for the first 2 h and then every 1 h. The release rate of the active substance was determined by UV-Vis measurements using a Varian Cary 50 Bio UV-Vis spectrophotometer. The characteristic wavelengths corresponding to the maximum absorbance of the used active substances in a given solvent were as follows: (a) ibuprofen: 272 nm; (b) curcumin: 425 nm; (c) ferulic acid: 319 nm. The percentage of the release of active ingredients from carriers was calculated with Equation (2) [45]:

(2) $$\%\;\mathrm{release}=\frac{A_{p}}{A_{w}}\left(\frac{m_{w}\left[\mathrm{mg}\right]{\;x\;C}_{w}}{V_{w}\left[\mathrm{ml}\right]}\right)\left(\frac{1}{D_{w}}\right)\left(\frac{V_{p}\left[\mathrm{ml}\right]}{m_{p}\left[\mathrm{mg}\right]}\right)\times 100\%$$

where: A_p_—absorbance of the sample (a.u.), A_w_—absorbance of standard (a.u.), m_w_—a mass of standard (mg), m_p_—a mass of active substance contained in the sample (mg), C_w_—purity of standard (a.u.), D_w_—dilution of standard (a.u.), V_w_—the volume of standard solution (ml), V_p_—the volume of acceptor fluid (mL).

Equation (2) is the formula to calculate the percent of release of active substances from the carrier [45].

### 2.6. Leaching Tests

Leaching tests for modifying agents (polysaccharides) were performed in the case of all the materials obtained. The following procedure was applied: 20 mg of polysaccharide-modified support was weighed in a glass vial, a mixture of 20.00 mL of phosphate buffer (pH 5.8) and 5.00 mL of glycerol (permeation promoter) was added. It was stirred for 24 h. After this time, materials were filtered, dried and characterized using elemental analysis.

## 3. Results

### 3.1. Elemental Analysis

To determine the exact qualitative composition of the hierarchical zeolites obtained, an elemental analysis was performed.

The percentages of nitrogen, carbon, hydrogen, and sulfur in the hierarchical zeolites synthesized, which were evaluated by means of elemental analysis, are shown in Table 2. It was observed that the percentages of nitrogen are significantly higher for zeolites modified with heparin and hyaluronic acid when compared to the pure hierarchical materials. This may be due to the fact that heparin and hyaluronic acid have nitrogen atoms in their structure. At the same time, it confirms the effectiveness of modification of the obtained materials with these molecules. Additionally, an increase in the percentage of C and H atoms was observed in comparison to that for commercial and hierarchical unmodified materials. This is due to the fact that during the synthesis of these materials modifying agents were used, i.e., inulin, heparin and hyaluronic acid which are organic molecules, giving the obvious rise in the content of C and H atoms. 

Leaching tests were carried out to verify whether leaching of modifiers (polysaccharides) into the acceptor fluid may occur during the release process. The results obtained on the basis of elemental analysis (Table 2) indicate that the content of C, H, N and S in the tested materials after 24 h of mixing with acceptor fluid (phosphate buffer + glycerol) does not change significantly. It can be estimated that leaching does not exceed 2%, which indicates that the molecules of the modifying agents are quite firmly bound to the zeolite matrix.

### 3.2. XRD—X-ray Diffraction 

One of the main methods for identifying nanometer-sized objects, such as self-organized structures, is X-ray diffraction (XRD). XRD is mainly used to determine the structure of the substance under study. Therefore, when testing the surface of substances, the XRD method complements the information obtained using transmission microscopy. It also enables the observation of phase transitions and catalytic reactions taking place on the tested surface. It is especially used in the case of heterostructures and multilayered systems, concerning layers of thickness up to nanometers. Obviously, XRD is not a technique that allows complete characterization of porous materials; therefore, to evaluate the physicochemical properties of the materials obtained based on commercial zeolites such as MFI (ZSM-5), BEA (β), and FAU (Y) in addition to the XRD method, other tests were also carried out.

**Figure 2:**
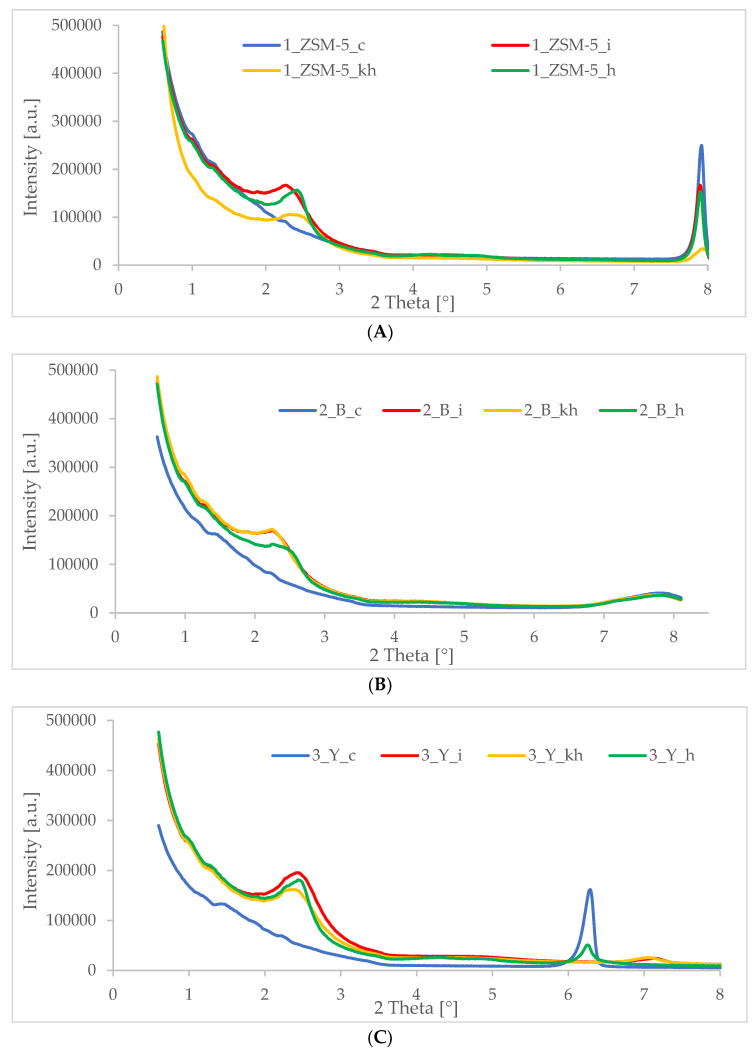
Diffractograms in low-angle range of hierarchical materials: (**A**) based on commercial zeolite of ZSM-5 type (1_ZSM-5_c—commercial zeolite, 1_ZSM-5_i—inulin modified material, 1-ZSM-5_kh—hyaluronic acid modified material, 1_ZSM-5_h—heparin modified material); (**B**) based on commercial zeolite of BEA type (2_B_c—commercial zeolite, 2_B_i—inulin modified material, 2_B_kh—hyaluronic acid modified material, 2_B_h—heparin modified material); (**C**) based on commercial zeolite of FAU type (3_Y_c—commercial zeolite, 3_Y_i—inulin modified material, 3_Y_kh—hyaluronic acid modified material, 3_Y_h—heparin modified material).

For the materials synthesized in this work, intense and broad reflections at 2θ~1.5–2.5° were recorded in all diffractograms taken in the low angle range (Figure 2A–C), which confirms the acquisition of additional mesoporous structure in the obtained catalysts. In the above-mentioned diffractograms in the low-angle range, the occurrence of additional reflections can also be observed, at an angle of 2θ < 2.5°, which indicates a more ordered structure of the obtained hierarchical zeolites. Similar results were described previously by Feliczak-Guzik and co-workers and Ramezani and co-workers [44,46]. Comparing the diffractograms of the materials before and after extraction, one can observe that the diffractograms of the materials after extraction are characterized by more intense (sharper/less stretched) reflections at the angle 2θ~1.5–2.5° The interplanar spacing is a result of the statistical distribution of geometrically disordered pores.

**Figure 3:**
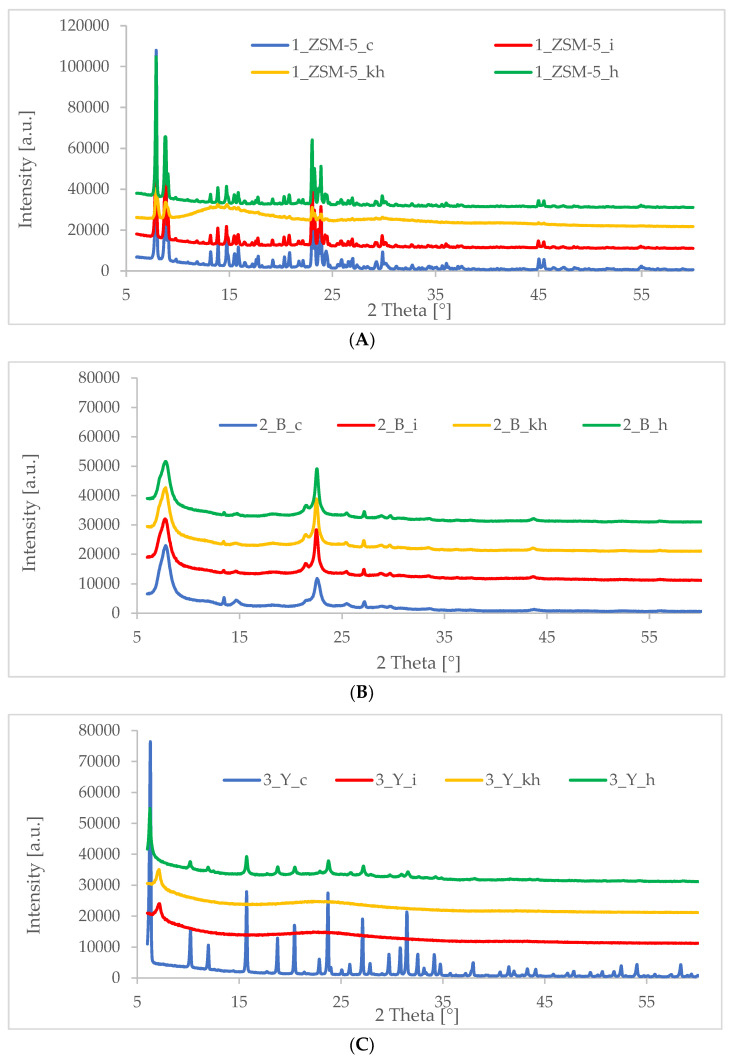
Diffractograms in wide-angle range of hierarchical materials: (**A**) based on commercial zeolite of ZSM-5 type (1_ZSM-5_c—commercial zeolite, 1_ZSM-5_i—inulin modified material, 1-ZSM-5_kh—hyaluronic acid modified material, 1_ZSM-5_h—heparin modified material); (**B**) based on commercial zeolite of BEA type (2_B_c—commercial zeolite, 2_B_i—inulin modified material, 2_B_kh—hyaluronic acid modified material, 2_B_h—heparin modified material); (**C**) based on commercial zeolite of FAU type (3_Y_c—commercial zeolite, 3_Y_i—inulin modified material, 3_Y_kh—hyaluronic acid modified material, 3_Y_h—heparin modified material).

On the other hand, the diffractograms in the high angle range obtained for the synthesized materials (Figure 3A–C) in most cases confirmed the preservation of the crystal structure of the used microporous commercial zeolites of MFI (ZSM-5), BEA (β), and FAU (Y) types. Only in the diffractogram obtained for hierarchical zeolites based on FAU zeolite modified with inulin and hyaluronic acid (Figure 3C) and for the material based on zeolite ZSM-5 modified with hyaluronic acid (Figure 3A), after the extraction process, it was noticed that the crystal structure of commercial zeolite was not preserved, indicating destruction of the crystal structure of the starting materials or the amorphization of the surface to extended levels resulting from the performed modification process.

In summary, all the low-angle diffractograms of the obtained catalysts, presented below, confirm the obtaining of additional secondary porosity (reflection 2θ~1.5–2.5°). This phenomenon is observed when the template is used to construct the ordered mesopores [47]. On the other hand, thanks to the diffractograms in the high angle range, the structure preservation of the starting commercial zeolites was confirmed in most cases.

### 3.3. Nitrogen Adsorption/Desorption Isotherms

From sorption studies, textural properties can be determined for newly synthesized hierarchical materials based on commercial zeolites such as MFI (ZSM-5), BEA (β), and FAU (Y), namely specific surface area, total pore volume, micropore volume, mesopore volume, average pore size, and pore size distribution.

On the basis of nitrogen adsorption/desorption isotherms (Figure S1 in Supplementary Materials) for commercial materials, viz: BEA (β) and FAU (Y), type I isotherms may be observed according to the International Union of Pure and Applied Chemistry (IUPAC), which is characteristic of microporous materials, was reported [48]. The commercial MFI (ZMS-5) zeolite is characterized by adsorption/desorption isotherms of mixed type I and IV, the latter being characteristic isotherm for mesoporous materials, which would indicate that it was not a typical microporous material already at the time of purchase.

In the case of the modified hierarchical materials, the presence of a mixture of isotherms, both type I and type IV, was noted. The type IV isotherm is characterized by the presence of three ranges:

- in the first one, there is a linear increase of adsorbed nitrogen while the pressure p/p_0_ has a low value; this correlates with monolayer adsorption deposited on the pore walls;
- in the second range, there is a rapid increase in adsorbed nitrogen for medium pressures p/p_0_, which is a capillary condensation effect occurring in the mesopores;
- in the third range, there is a gradual, linear increase in p/p_0_ in the high-pressure region, which results in nitrogen adsorption on the outer surface of the material, i.e., in the spaces between the pores.

The obtained results confirm the obtaining of hierarchical materials with secondary porosity, in the mesopore range. It can also be observed that, depending on the type of polysaccharide used to modify the pure zeolite, there is a slightly different increase in the total amount of adsorbed nitrogen, which may indicate an increase in the textural properties. For modified materials in the high relative pressure region, a rapid increase in adsorbed nitrogen can also be observed, indicating the formation of a secondary porosity called textural [49,50]. Similar results were described earlier by Ramezani and co-workers [46].

The very narrow pore size distribution is characteristic for commercial microporous materials of type MFI (ZSM-5), BEA (β), and FAU (Y). For all three types of materials, the largest and virtually the only pore size distribution is for pores with a width of 0.5 nm, which is within the size limit assigned to microporous materials. A narrow pore size distribution is also evident for synthesized polysaccharide-modified materials, but the pore widths for inulin, heparin, and hyaluronic acid modified materials range from about 2.5 nm to about 12 nm. For hierarchical materials based on MFI type zeolite (ZSM-5) modified with hyaluronic acid and inulin a larger pore size distribution can be observed, with a pore width of about 2.5 nm compared to heparin modified materials. A large pore size distribution can also be observed for inulin-modified materials, with a pore size of about 8.0 nm. On the other hand, for the materials based on zeolite BEA (β), the distribution of the pore size is similar for the three modified materials. For the materials based on the FAU (Y) zeolite, the pore size distribution varies from about 2.5 nm to 4.0 nm.

The selected textural properties of the hierarchical materials based on the commercial zeolites MFI (ZSM-5), BEA (β), and FAU (Y) are shown in Table 3.

The polysaccharide-modified and unmodified hierarchical zeolites exhibit properties typical of materials having porosity in the mesopore range. These include:

- high specific surface area, which varies from about 400 to 830 [m^2^/g];
- high porosity, in which the total pore volume is as high as 0.66 [cm^3^/g];
- homogeneous pore size, where the pore width ranges from 3.0 to 3.5 nm.

Unmodified materials had the largest specific surface area (Table 3). Polysaccharide-modified materials, on the other hand, showed similar specific surface area, ranging from 405 to 641 m^2^/g. The smallest total pore volume, and the largest micro pore volume, was observed for commercial materials such as MFI (ZSM-5), BEA (β), and FAU (Y). However, for the materials synthesized in this study, the presence of a high total pore volume, low micropore volumes, and increased mesopore volumes was observed compared to the initial microporous materials, which indicates that a hierarchical structure of the obtained materials was obtained.

In conclusion, based on the textural properties of the materials obtained (Table 3), the presence of secondary porosity (mesoporosity) can be confirmed for both polysaccharide-modified and unmodified materials.

### 3.4. Transmission Electron Microscopy (TEM)

Images taken by transmission electron microscopy (TEM) for polysaccharide-modified materials are shown in Figure S2 (supporting information). For all the materials obtained, it can be observed that all the oval-shaped particles are very loose and porous. These particles, 10–20 nm in size, are grouped into irregularly shaped clusters ranging from 100 to 400 nm in size. 

### 3.5. Active Substances Loading and Release Profiles

The active substances loading into hierarchical zeolites was calculated using Equation (1). The results of the analysis of ibuprofen, curcumin, or ferulic acid loading in the zeolite carrier are included in Table 4. 

The loading percentage of all active substances ranges from 39–79%. The loading percentage of ibuprofen and ferulic acid in a given carrier depends on the specific surface area of the material and its mesoporous volume, that is, the higher the specific surface area and the mesoporous volume, the higher the loading percentage. However, that is not the case for curcumin, where the loading percentage is quite similar for each carrier tested, regardless of its textural parameters. This may be caused by the steric hindrance created by the curcumin molecule, which is the largest molecule among all active substances tested and thus it may be difficult for it to penetrate deeper regions of pores. Furthermore, it may be observed that when comparing ibuprofen and ferulic acid, in most cases higher values of % LOAD were obtained for the latter molecule. An explanation for this may be the highest polarity of ferulic acid among all molecules tested by us, for which the logP is 1.57, while for ibuprofen and curcumin the value of this parameter is 3.97 and 3.29, respectively. The trend due to differences in the polarity of the active substance molecules is most evident in the case of unmodified zeolites, while for polysaccharide-functionalized carriers it becomes somewhat less apparent, probably due to the additional influence of the steric factors induced by polysaccharide molecules.

During presented studies the release profiles of three active substances, such as ibuprofen, curcumin, and ferulic acid from polysaccharide-modified hierarchical zeolites into the acceptor fluid (phosphate buffer, pH 5.8) were evaluated. Acceptor fluid, also called medium, is a liquid into which transmembrane diffusion of the active substance from the formulation under study takes place. The selection of the acceptor fluid should be guided by the measure of its similarity to the physiological conditions (temperature, pH value) of the skin. Note that the pH value of healthy skin is in the range of 4.5–6.5 [51]. Therefore the phosphate buffer with pH 5.8 was selected during our studies.

Additionally, glycerin was used as a permeation promoter and in the case of ferulic acid also ethyl alcohol.

Glycerol and ethanol were used as permeation promoters. These are chemicals that increase the penetration of the active ingredient through the skin. They can increase permeation of active substances by removing the hydrolipidic layer and partially dissolving the lipids of the intercellular cement of the epidermis [52,53,54]. To determine the release profile of ferulic acid two permeation promoters were used, since satisfactory results were not obtained in the glycerin-infused acceptor fluid. 

UV–Vis spectrophotometry enabled the determination of the profile of release of bioactive substances from the carrier into the acceptor medium. The result of the release of the active substance from hierarchical materials is a change in its concentration in the acceptor solution; hence the amount of the released active substance may be assessed by measuring the change in absorbance over time, according to the Lambert-Beer law. In addition, the release process was conducted using a multipoint assay, which provided information on changes in the amount of released active substance at several time points.

**Figure 4:**
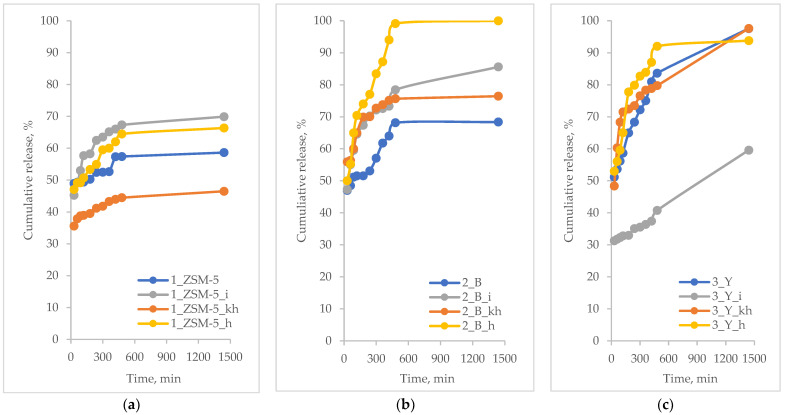
Percentage of ibuprofen released from modified hierarchical zeolites; (**a**) samples based on ZSM-5 zeolite; (**b**) samples based on BEA zeolite; (**c**) samples based on FAU zeolite.

**Figure 5:**
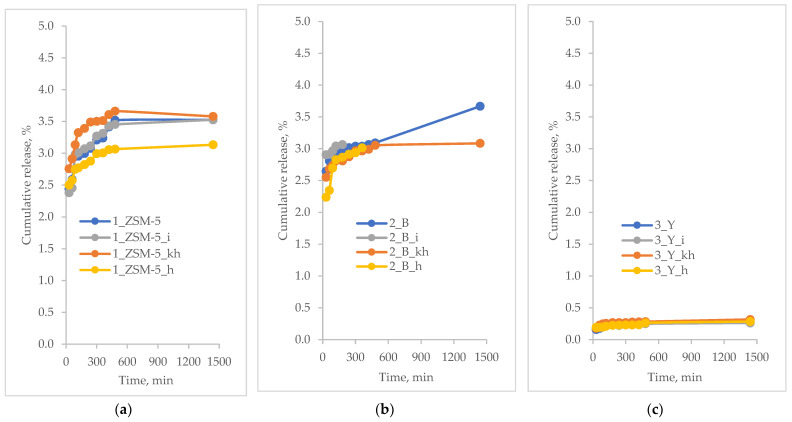
Percentage of curcumin released from modified hierarchical zeolites; (**a**) samples based on ZSM-5 zeolite; (**b**) samples based on BEA zeolite; (**c**) samples based on FAU zeolite.

**Figure 6:**
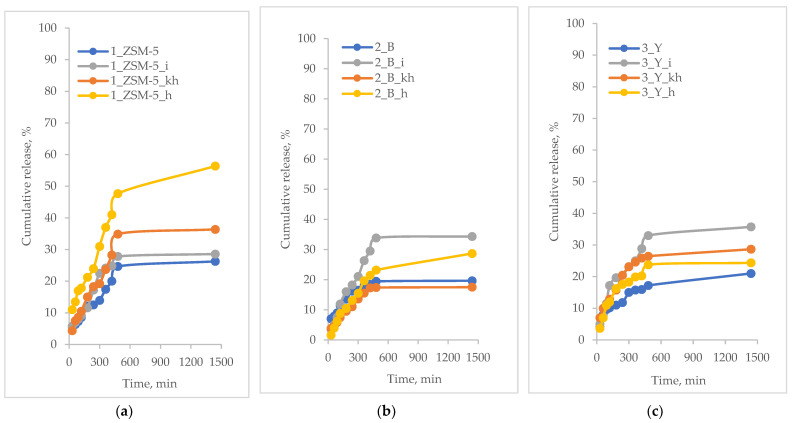
Percentage of ferulic acid released from modified hierarchical zeolites into acceptor fluid with glycerin; (**a**) samples based on ZSM-5 zeolite; (**b**) samples based on BEA zeolite; (**c**) samples based on FAU zeolite.

**Figure 7:**
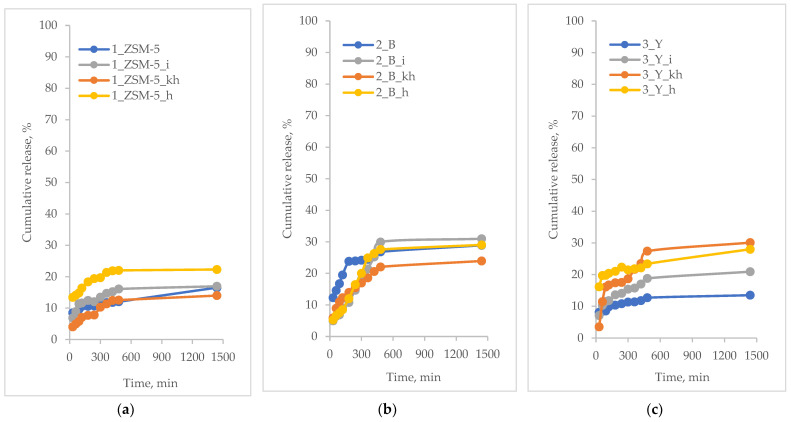
Percentage of ferulic acid released from modified hierarchical zeolites into acceptor fluid with ethanol; (**a**) samples based on ZSM-5 zeolite; (**b**) samples based on BEA zeolite; (**c**) samples based on FAU zeolite.

The in vitro release profiles of the active substance are presented as a time-dependent curve of the percentage release of the active substance. The results of the controlled release of the active substances from hierarchical zeolites based on commercial zeolites are shown in Figure 4, Figure 5, Figure 6 and Figure 7.

Based on the results of the release of ibuprofen from hierarchical zeolites (Figure 4), it can be concluded that all polysaccharide-modified materials showed higher release rates compared to unmodified materials. Previously, the same tendency was observed after the modification of the surface with amine groups, i.e., the structure of mesoporous molecular sieve of SBA-16-type modified with chitosan significantly affects the release rate of furosemide [55]. A correlation was observed between the release rate of ibuprofen and the type of polysaccharide used during the synthesis of hierarchical zeolites. The sample of BEA zeolite modified with heparin (2_B_h, surface area 611 m^2^/g and total pore volume of 0.46 cm^3^/g), showed the fastest ibuprofen release rate of 100 % after 8 h, while the release rate from the other materials depended on the structure of the commercial zeolite used during the preparation of the hierarchical zeolite. The trend in percentage release of ibuprofen from the zeolite used indicated a positive influence of heparin and inulin molecules, and was as follows:

- MFI-based hierarchical zeolite: 1_ZSM-5_i; 1_ZSM-5_h; 1_ZSM-5; 1_ZSM-5_kh
- BEA-based hierarchical zeolite: 2_B_h; 2_B_i; 2_B_kh; 2_B
- FAU-based hierarchical zeolite: 3_Y_h; 3_Y; 3_Y_kh; 3_Y_i

Furthermore, it was observed that the release of ibuprofen proceeded in two stages; some ibuprofen molecules were adsorbed on the outer surface of the materials. Their release occurred early in the course of the process. In contrast, molecules that were inside the pores of the hierarchical zeolites were slowly released into the acceptor fluid. Besides, the highest release rate for most materials was obtained for the heparin-modified samples. This suggests that the ibuprofen molecules in these materials were mainly adsorbed on their external surface, making them more accessible [56]. Moreover, heparin molecule is the smallest one among polysaccharides used, thus it does not block significantly the loading and releasing of the active substance from the pores of zeolites.

A large number of silanol groups located on the surface of the mesopores and at the entrance to micropores make zeolitic materials heterogeneously active in the interactions with the “guest” molecules. In this case the molecules of the active substance, i.e., ibuprofen, preferentially deposit inside the mesopores and at the entrance of micropores in the form of a thin film [57,58].

The curcumin release profiles of from modified hierarchical materials have been shown in Figure 5.

As observed, the release percentage of curcumin from the carriers under study is less than 5%. Such a low value may be due to the fact that the relatively large curcumin molecule, once deposited in the pores of the carriers, may have trouble desorbing from inside the pores. The peculiar behavior of the curcumin molecule could already be observed during the measurement of the loading percentage. The degree of this parameter was significantly lower than that of other active substances (Table 4). The obtained release profiles (Figure 5) may indicate that curcumin molecules, once deposited in the pores, become blocked due to a certain steric hindrance. In addition, it can also be observed that for MFI-based and BEA-based hierarchical zeolites the release percentage is slightly higher than for FAU-based zeolite. This may be due to the fact that in the case of the latter carrier, which has the highest proportion of mesopores (mesopore size: 3.4 nm, Table 3), during the introduction of curcumin, the molecules of this compound may have diffused deeper into the pore system and then may have been blocked there, also by the introduced polysaccharides. On the other hand, in the case of carriers with smaller mesopore sizes, that is, 3.0 and 3.2 for MFI-based and BEA-based zeolites, respectively (Table 3), due to the more difficult accessibility of the deeper portions of the pores, the curcumin adsorption may have occurred to some extent on the carrier surface and in the pore inlets. It is likely that this part of the loaded curcumin is first released in the case of these two zeolite materials (Figure 5b). Therefore, the release of curcumin from modified hierarchical zeolites based on commercial zeolites of MFI and BEA-type showed an initial burst-type release within the first 30 min of the process. A similar relationship was not observed for materials derived from the FAU-type commercial zeolite. This type of release may be related to the surface-bound curcumin molecules on the surface of the carriers used [59].

Ferulic acid release studies were carried out using acceptor fluid at pH 5.8 with the addition of two different permeation promoters, that is, ethanol or glycerol. The percentage of ferulic acid released from the modified hierarchical zeolites is pictured in Figure 6 (glycerin) and Figure 7 (ethanol).

## 4. Conclusions

In the present work, unmodified and modified hierarchical materials with polysaccharides such as inulin, hyaluronic acid, and heparin, with secondary porosity (hierarchical zeolites), based on commercial materials such as MFI (ZSM-5), BEA (β) and FAU (Y) were obtained. Thorough physicochemical characterization of all the hierarchical materials obtained was carried out. In addition, they were also applied as platforms for controlled loading and release of active ingredients with anti-inflammatory activity, namely ibuprofen, curcumin, and ferulic acid. It has been proved that the degree and rate of release of the active substance depends on the matrix of the porous material (the highest percentage of release, close to 100%, was obtained for ibuprofen loaded on heparin-modified BEA-based hierarchical zeolite). The introduction of polysaccharides containing extra OH groups into zeolitic structures allows for acquisition of additional sites available for binding molecules of active substances, as shown during our studies. In most cases, polysaccharide-modified zeolites seem to be a better platform for drug delivery than their unmodified counterparts. Significant influence of the type of the polysaccharide on the loading and release profile of curcumin, ibuprofen, and ferulic acid has also been demonstrated: in most cases heparin seems to be the most influenceable modifying agent.

## Figures and Tables

**Table 1:** Designation of materials used in the work.

X_M		
X—STRUCTURE	M—MODIFICATION	DESCRIPTION
MFI	Commercial zeolite	1_ZSM-5_c
MFI	Unmodified hierarchical zeolite	1_ZSM-5
MFI	Inulin modified hierarchical zeolite	1_ZSM-5_i
MFI	Hyaluronic acid modified hierarchical zeolite	1_ZSM-5_kh
MFI	Heparin modified hierarchical zeolite	1_ZSM-5_h
BEA	Commercial zeolite	2_B_c
BEA	Unmodified hierarchical zeolite	2_B
BEA	Inulin modified hierarchical zeolite	2_B_i
BEA	Hyaluronic acid modified hierarchical zeolite	2_B_kh
BEA	Heparin modified hierarchical zeolite	2_B_h
FAU	Commercial zeolite	3_Y_c
FAU	Unmodified hierarchical zeolite	3_Y
FAU	Inulin modified hierarchical zeolite	3_Y_i
FAU	Hyaluronic acid modified hierarchical zeolite	3_Y_kh
FAU	Heparin modified hierarchical zeolite	3_Y_h

**Table 2:** Elemental analysis of hierarchical materials obtained from zeolites of MFI (ZSM-5), BEA (β), and FAU (Y) types.

Material	%N	%C	%H	%S
1_ZSM-5_c	0.123	0.742	0.349	0.073
1_ZSM-5	0.041	0.023	0.908	0.004
1_ZSM-5_i	0.120	3.095	4.088	0.425
1_ZSM-5_i (leaching)	0.117	3.086	4.075	0.418
1_ZSM-5_kh	0.088	3.772	1.125	0.335
1_ZSM-5_kh (leaching)	0.086	3.705	1.105	0.320
1_ZSM-5_h	0.207	4.633	1.348	0.494
1_ZSM-5_h (leaching)	0.205	4.601	1.302	0.487
2_B_c	0.095	0.080	1.333	0.045
2_B	0.014	0.059	0.655	0.261
2_B_i	0.278	6.589	2.124	0.368
2_B_i (leaching)	0.208	6.498	2.012	0.302
2_B_kh	0.271	6.781	2.176	0.340
2_B_kh (leaching)	0.206	6.691	2.087	0.328
2_B_h	0.341	7.535	2.298	0.140
2_B_h (leaching)	0.332	7.451	2.176	0.132
3_Y_c	0.011	0.024	2.836	0.273
3_Y	0.014	0.011	2.582	0.303
3_Y_i	0.018	2.652	2.709	0.140
3_Y_i (leaching)	0.017	2.543	2.602	0.131
3_Y_kh	0.022	3.066	2.957	0.027
3_Y_kh (leaching)	0.018	2.990	2.876	0.022
3_Y_h	0.144	3.280	3.526	0.108
3_Y_h (leaching)	0.140	3.214	3.455	0.104

**Table 3:** Textural parameters of hierarchical zeolites prepared from commercial materials of ZSM-5, BEA (β), and FAU (Y) types.

Material	Specific Surface Area, BET (m^2^/g)	Pore Volume, (cm^3^/g)			Mesopore Size, (nm)
		Total Pore Volume	Micropores Volume	Mesoporous Volume	
1_ZSM-5_c	347	0.26	0.13	0.13	3.0
1_ZSM-5	829	0.57	0.23	0.34	2.7
1_ZSM-5_i	422	0.56	0.16	0.40	3.1
1_ZSM-5_kh	405	0.35	0.15	0.20	3.5
1_ZSM-5_h	445	0.40	0.13	0.27	3.3
2_B_c	533	0.29	0.25	0.04	-
2_B	704	0.42	0.18	0.24	3.2
2_B_i	440	0.54	0.12	0.42	3.1
2_B_kh	529	0.36	0.17	0.19	3.1
2_B_h	611	0.46	0.17	0.29	3.0
3_Y_c	718	0.37	0.34	0.03	-
3_Y	792	0.49	0.19	0.30	3.4
3_Y_i	610	0.66	0.22	0.44	3.1
3_Y_kh	604	0.47	0.23	0.24	3.0
3_Y_h	641	0.61	0.18	0.43	3.3

**Table 4:** Active substances loading into hierarchical zeolites.

Materials	% LOAD		
	IBUPROFEN 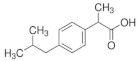	CURCUMIN 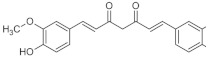	FERULIC ACID 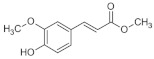
1_ZSM-5	49	39	70
1_ZSM-5_i	68	39	49
1_ZSM-5_kh	50	39	54
1_ZSM-5_h	43	39	44
2_B	54	38	64
2_B_i	46	40	58
2_B_kh	49	40	47
2_B_h	53	39	44
3_Y	54	44	62
3_Y_i	43	41	48
3_Y_kh	79	42	50
3_Y_h	53	40	50

## Data Availability

Not applicable.

## Supplementary Materials

The following supporting information can be downloaded at: https://www.mdpi.com/article/10.3390/pharmaceutics15020535/s1, Figure S1: Nitrogen adsorption/desorption isotherms for materials based on commercial zeolites of ZSM-5, BEA, and FAU types; Figure S2: Transmission electron microscopy (TEM) images of hierarchical zeolites modified with polysaccharides.
