# Supplementary material for: Synthesis and Characterization of Hierarchical Zeolites Modified with Polysaccharides and Its Potential Role as a Platform for Drug Delivery
Source: Pharmaceutics. 2023 Feb 5;15(2):535. doi: 10.3390/pharmaceutics15020535 (PMC9968069; doi:10.3390/pharmaceutics15020535)
Supplement: Supplementary file 1 [file pharmaceutics-15-00535-s001.zip › pharmaceutics-2174432-supplementary.pdf]

Supplementary Materials: Synthesis and Characterization of Hierarchical Zeolites Modified with Polysaccharides and Its Potential Role as a Platform for Drug Delivery

Agata Wawrzyńczak, Izabela Nowak, Natalia Woźniak, Jagoda Chudzińska, and Agnieszka Feliczak-Guzik

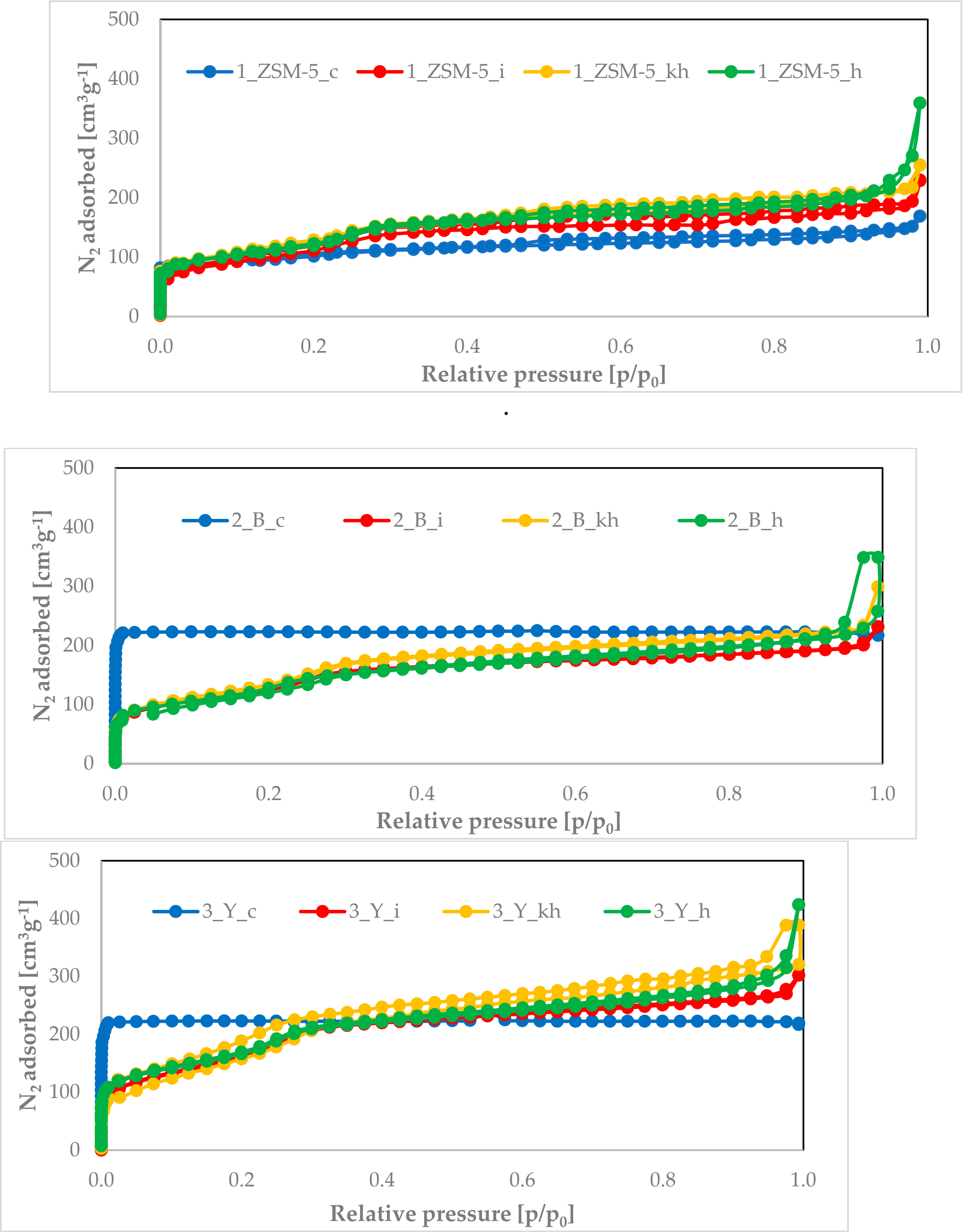

(A)

(B)

(C)

Figure S1. Nitrogen adsorption/desorption isotherms for materials: **(A)** based on commercial zeolite of ZSM-5 type (1_ZSM-5_c – commercial zeolite, 1_ZSM-5_i – inulin modified material, 1-ZSM-5_kh - hyaluronic acid modified material, 1_ZSM-5_h - heparin modified material); **(B)** based on commercial zeolite of BEA type (2_B_c –

commercial zeolite, 2_B_i – inulin modified material, 2_B_kh - hyaluronic acid modified material, 2_B_h - heparin modified material); (C) based on commercial zeolite of FAU type (3_Y_c – commercial zeolite, 3_Y_i – inulin modified material, 3_Y_kh - hyaluronic acid modified material, 3_Y_h - heparin modified material).

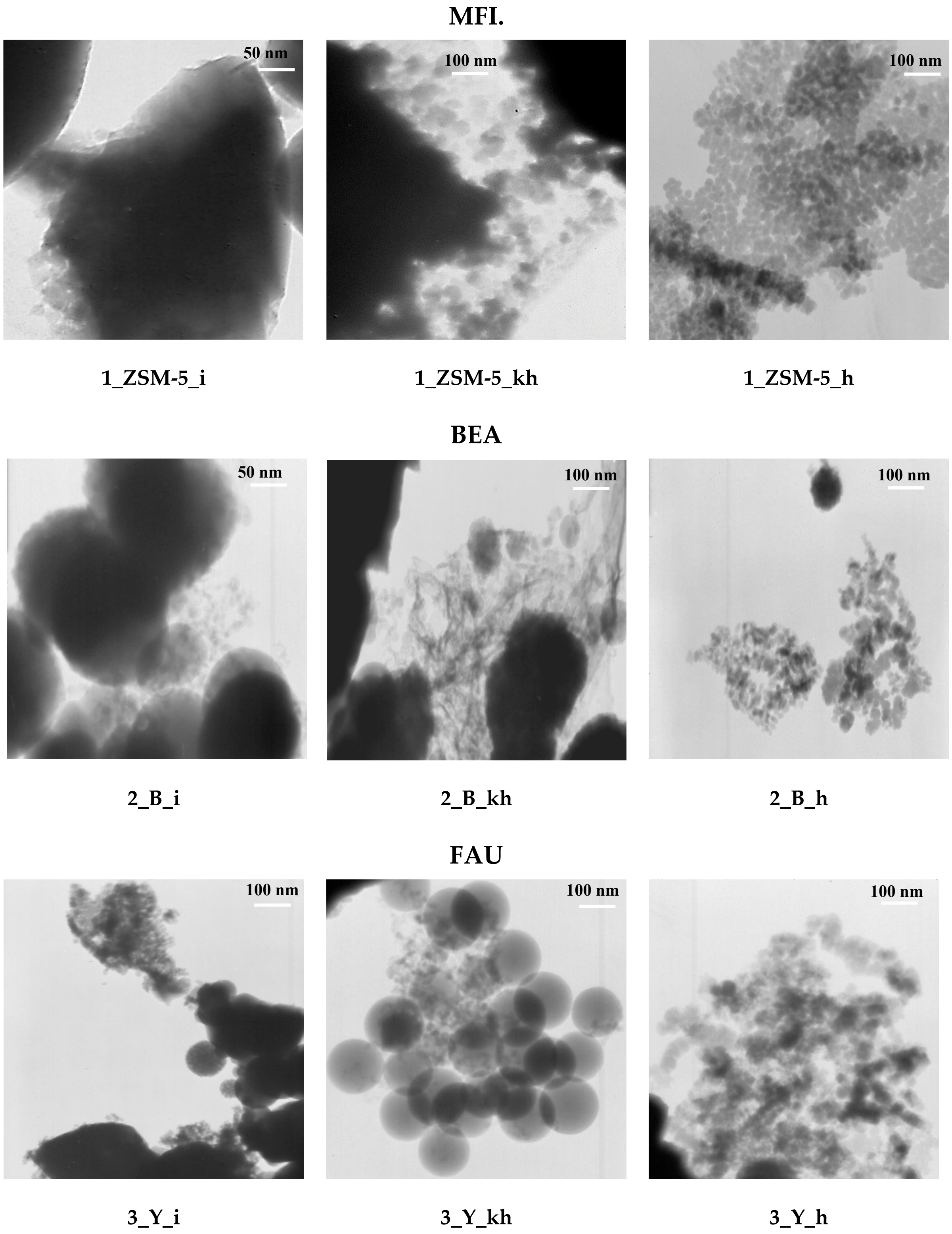

Figure S2. Transmission electron microscopy (TEM) images of hierarchical zeolites modified with polysaccharides.
